# Titanium dioxide nanoparticles on flexural strength of conventional and injection moulded denture base materials

**DOI:** 10.6026/973206300210019

**Published:** 2025-01-31

**Authors:** Udayagiri Madhusudhana Rao, Nibha Kumari Singh, Seemala Jyotsna, Poojitha Y., Ravada V SSK Kinneresh, Adithya Teja Prasad Pallekonda

**Affiliations:** 1 Department of Posthodontics Crown and Bridge, Government Dental College, Kadapa andhra Pradesh, India; 2 Department of Public Health Dentistry, Government Dental College, Kadapa andhra Pradesh, India; 3 Department of Posthodontics Crown and Bridge, Narayana Dental College and Hospital, Nellore andhra Pradesh, India; 4 Department of Public Health Dentistry, Meghana Institute of Dental Sciences, Nizamabad, Telangana, India

**Keywords:** Titanium dioxide, nanoparticles, compression, flexural strength, optimization

## Abstract

The effect of anatase titanium dioxide nanoparticles on the flexural strength of poly-methyl-methacrylate (PMMA) resins comparing conventional and injection molding techniques is of interest. Hence, a total of 120 acrylic specimens were prepared and divided into control, 0.5% TiO_2_ and 1% titanium dioxide groups. Flexural strength was measured using a three-point bend test and analyzed using variance test. The injection molded group showed superior flexural strength compared to the compression molded group with control specimens exhibiting the highest values. Increasing titanium dioxide concentration led to a significant reduction in flexural strength indicating a negative correlation between nanoparticle content and mechanical performance.

## Background:

Acrylic resins have been modified to improve not only their physical and chemical properties but also their working properties in the processing of complete dentures [1]. To address dimensional inaccuracies in conventional compression molding, Pryor adapted an injection molding technique for acrylic resin, using hydraulic pressure to compensate for shrinkage. However, Grunewald *et al.* found no significant advantages and Pryor's method didn't gain popularity. Later, in the mid-1970s, Ivoclar introduced a modified acrylic resin for injection molding, which became popular as other dental companies adopted similar systems, offering improved accuracy and efficiency [2]. Injection molding allows directional control of the polymerization process through the flask design. A constant flow of new material from the sprue compensates for the polymerization shrinkage [3, 4-5]. Nanoparticles as additives can alter the physical properties of the substrates. Among resin based materials nano-TiO_2_ has been added in various proportions to composites, to enhance their special characteristics and its effects on overall mechanical properties is mentioned. Moreover incorporation of Ti, Zn, or Ce nano-oxides to commercial silicone elastomer has been shown to improve their tensile and tear strength and percent elongation [6]. Therefore, it is of interest to evaluate how different concentrations of anatase TiO_2_ nanoparticles affect the flexural strength of poly-methyl-methacrylate resins, processed by conventional and injection molding techniques, to ensure no detrimental effects reduce strength below the standard limit.

## Materials and Methods:

A total of 120 acrylic specimens (50x10x3 mm) were prepared according to ISO 20795-1(2008) standards, with 60 processed using compression molding and 60 using injection molding. The specimens were divided into three groups: a control group (Nc) without TiO_2_ nanoparticles and two experimental groups with 0.5% (N0.5) and 1% (N1) anatase phase TiO_2_ nanoparticles (average diameter 56nm). In the present study titanium dioxide nanoparticles were incorporated into the compression molded and injection molded acrylic resin. The nanoparticles were added to the acrylic monomer at concentrations of 0.02g/ml and 0.04g/ml to achieve the desired percentages. Main purpose of the study was to evaluate the effect of titanium dioxide nanoparticles incorporation on the flexural strength of these two resins. Flexural strength values were measured using three point bend test. The two heat cure acrylic resins that were used in the present study were conventional heat cure (DPI, MUMBAI) and injection molded arylic resin (SRIVOCAPHIGH IMPACT, IVOCLAR, VIVADENT). The study was carried out fewer than two groups and six sub groups. Grouping was done based on type of material used and processing technique. Sub grouping was done based on the concentration of TiO_2_NPs 0%, 1% and 5% concentrations of nanoparticles. Specimens were fabricated according to ISO20795-1(2008). Specimens were divided into two groups namely N and injection molded. These two groups were subdivided into six sub groups (3 sub-groups per each group). Six subgroups were Nc, N1, N5, IMc, IM1 and IM5. A total of 120 samples were fabricated (20samples per each group. Samples belongs to N group were processed by conventional compression molding technique and samples of injection molded group were processed by injection molded technique. Depending on the concentration of titanium dioxide nanoparticles they were subdivided into three sub groups they were, 1 and 5 which contain so %, 1% and 5% nanoparticles respectively. The specimens were finished and polished conventionally. The specimens were stored in distilled water for 50±2 hours prior to testing to simulate oral environment. Flexural strength of these specimens was tested using three point bend test on universal testing machine.

## Steps in the preparation of study samples:

## Preparation of metal die:

Five customs made Metal dies of dimensions 50 x 10 x 3 mm were fabricated.

## Preparation of monomer:

Titanium dioxide (TiO_2_) nanoparticles were incorporated into the monomer at concentrations of 0.5% and 1% by adding 0.02g and 0.04g of TiO_2_ NPs per 1ml of monomer, respectively. The nanoparticles were mixed into the monomer using ultrasonic dispersion for 30 minutes. The monomer, part of the injection-molded resin SR IVOCAP, was removed from its capsule and mixed with TiO_2_ nanoparticles to achieve the desired concentrations: 0.6g for 0.5% and 1.2g for 1% concentration.

## Compression molding technique:

Sixty samples were prepared using compression molding, divided into three groups: control (NC) without nanoparticles and two experimental groups with 0.5% (N0.5) and 1% (N1) titanium dioxide nanoparticles. Metal dies were invested in dental flasks, de-waxed and washed. Acrylic resin was packed into the molds and heat polymerization was carried out in a curing unit. The specimens were finished and polished. The experimental groups used monomer with 0.5% and 1% nanoparticles, achieved by adding 0.02 and 0.04g of TiO_2_ nanoparticles per ml of monomer. A curing cycle of 700°C for 1 hour and 950°C for 30 minutes was followed.

## Injection moulded resin processing:

A sixty injection-molded resin samples (SR IVOCAP) were prepared, divided into three groups: control (IMc), 0.5% titanium dioxide nanoparticles (IM0.5) and 1% nanoparticles (IM1). Each sample was fabricated using a standardized investment procedure, followed by injection molding with the respective monomer-nanoparticle mixtures. The samples were then polymerized in a water bath, cooled and deflasked using a hydraulic press. Finally, the retrieved samples were finished and polished. This rigorous process ensured uniformity and consistency across all samples.

## Flexural strength testing:

Prior to flexural strength testing, the specimens were stored in 37°c distilled water for 50 ± 2 h to simulate oral environment. The acrylic specimens were inserted in universal testing machine for three-point bending test. The distance between the support arms was kept constant at 40 mm. The initial applied force was zero followed by gradual increase with the rate of 5 mm/min until the specimen fractured. The flexural load was measured at the point of fracture of the specimen in Newtons. The ultimate flexural strength was measured using the following formula:

Flexural strength q= 3FI/2bh^2^

F = maximum applied force in newton,

I= distance between the support arms,

b = width of the specimen,

h = height of the specimen prior to water storage.

I, b and h values were 40, 10 and 3 mm respectively.

## Finishing of the samples:

The samples were finished with a lathe-mounted 30 fluted fine cross cut tungsten carbide bur. The tungsten carbide bur was used to remove gross irregularities and surface nodules, thereby producing a smooth and smeared surface on heat-cured acrylic resin. Final finishing was done with lathe mounted 320, 400 and 600-grit sandpaper and rubber points at 3000 rpm for 90 seconds. Thus the standardized finishing protocol was followed for all 120 specimens. After final finishing, the samples were subjected to three polishing agents.

## Polishing of the samples:

All the 120 samples were polished by conventional polishing technique, *i.e.* pumice mixed with water in the ratio 1:1 by volume for 40 seconds at 3000 rpm.

## Statistical analysis:

Statistical analysis was carried out using SPSS 20 software package (IBM Company). The mean flexural strength of each group was analyzed with analysis of variance test (Anova). Statistical significance was set at p<0.05. Descriptive statistical analysis has been carried out in the present study. Results of the test were presented on mean ±SD, analyzed using analysis of variance test.

## Results:

Flexural load values that were obtained from three point bend test were as given in Table 1. The injection molded (IM) group exhibited higher flexural load values compared to the compression molded (N) group. Notably, within each group, the control group demonstrated the highest flexural load values, indicating that the absence of any modifications or additives resulted in the strongest material performance.

Injection molded group (IM group) had higher flexural load values compared to compression molded group (N group). In each group control group had highest flexural load values. Table 2 shows flexural strength values. A significant change in flexural strength was observed in both groups; with the control groups (Nc and IMc) exhibiting the highest mean flexural strength compared to the test groups. Specifically, the injection molded group had the highest flexural strength, with a mean value of 119±8.859. However, as the concentration of titanium dioxide nanoparticles increased, a decrease in flexural strength was observed in both groups, suggesting a negative correlation between nanoparticle concentration and flexural strength.

Table 3 shows analysis of variance test representing significant results. A significant change in the flexural strength was observed in both the groups. Mean flexural strength of control group (Nc, IMc) had highest flexural strength than the test group. Between the two groups injection molded group had highest flexural strength (119±8.859). Decrease in the flexural strength was observed with increase in the concentration of titanium di oxide nanoparticles in both the groups.

## Discussion:

In the present study, mean flexural strength of Nc sub group was 97.77 and 93.9, 86.6 for N1 and N5 subgroups respectively. Mean flexural strength for IMc, IM1 and IM5 was 119.2, 112.4 and 101.1 respectively. Chemical alteration of the poly-methyl-methacrylate might be the reason for higher flexural strength of injection molded resin. Incorporation of titanium dioxide nanoparticles has decreased the flexural strength of poly methyl methacrylate. Titanium dioxide nanoparticles acts as impurities within the matrix of poly-methyl-methacrylate and Titanium dioxide nanoparticles are tend to agglomerate with in the matrix of poly methyl methacrylate. These are as weakens the matrix of poly methyl methacrylate. This could the reason for the decrease in the flexural strength of poly-methyl-methacrylate with increase in the concentration of the titanium dioxide nanoparticles. Our results are comparable to study by carried out by Nazrikar *et al.* [7] that investigated the effect of titanium dioxide nanoparticles on the flexural strength of denture base resin. They found that adding 0.5% and 1% anatase titanium dioxide nanoparticles (7nm diameter) decreased the flexural strength of the resin. The nanoparticles acted as impurities within the acrylic resin matrix, creating weak areas that reduced the overall strength of the material. The study suggested that increasing the concentration of nanoparticles further decreased the flexural strength of the denture base resin. Another a like study by Ahmed *et al.* [8] found that adding titanium dioxide nanoparticles (46nm diameter) at concentrations of 1% and 5% decreased the flexural strength of both types of resins. The study concluded that there is an inverse proportional relationship between the concentration of titanium dioxide nanoparticles and the flexural strength of the resins, meaning that as the concentration of nanoparticles increases, the flexural strength decreases. This suggests that the addition of titanium dioxide nanoparticles compromises the mechanical properties of the denture base resins. Cevik *et al.* [9] found that adding 1% or 5% silica or prepolymer to acrylic resin decreased its flexural strength compared to the control group. SEM images revealed voids at fracture sites, suggesting that porosity in the silica groups may be responsible for the reduced strength. Surface roughness values were also higher in the silica groups, indicating porous surfaces. Notably, 1% silica showed higher surface roughness than 5% silica, suggesting that even small amounts of silica can negatively impact the resin's properties. On the contrast, a study by Harini *et al.* [10] investigated the effect of titanium dioxide nanoparticles on the flexural strength of Poly-methyl-methacrylate (PMMA) and found a significant increase in flexural strength with the incorporation of these nanoparticles. This improvement is attributed to the reduced filler size, which enhances fracture resistance and the functionalized nanoparticles' ability to bind to the polymer matrix, increasing adhesion and thereby augmenting the mechanical properties of the acrylic resin. This study suggests that the addition of titanium dioxide nanoparticles can actually enhance the flexural strength of Poly methyl methacrylate, contradicting previous findings that showed a decrease in strength. Safi [11] found that modifying poly-methyl-methacrylate with TiO_2_ NPs affects its thermal and mechanical stability, leading to decreased thermal expansion, contraction and E-Modulus, as well as reduced flexural strength and toughness. However, incorporating silanized TiO_2_ NPs improved impact strength, transverse strength and surface hardness, while reducing water sorption and solubility. Additionally, apatite-coated TiO_2_ NPs showed antifungal properties, inhibiting Candida adhesion and barium titanate (BaTiO3) addition made the composite thermally stable but increased its density, affecting denture retention. Gad *et al.* (2016) [12] found that incorporating Nano-ZrO2 into poly-methyl-methacrylate denture base resin significantly improved its mechanical properties. The study highlighted the importance of the resin/filler interface adhesion in enhancing the properties of the PMMA/nano-ZrO2 composite, suggesting that a strong bond between the resin and nano-ZrO2 particles is crucial for achieving optimal mechanical performance. The increase in flexural strength of the repaired denture base resin can be attributed to several factors, including the small size and uniform distribution of nano-ZrO2 particles, the silanization process and the surface design of the joint. Additionally, the transformation of ZrO2 from tetragonal to monoclinic phase, known as transformation toughening, absorbed energy and arrested crack propagation by placing the crack under compressive stress. SEM analysis revealed that the good distribution of nano-sized particles and interstitial filling of the acrylic resin matrix with ZrO2 interrupted crack propagation, leading to enhanced flexural strength.

Hamedi-Rad *et al.* (2014) [13] found that adding 5-20% aluminium oxide powder (Al2O3) to heat-polymerized acrylic resin increases its flexural strength and thermal conductivity. In contrast, adding silica to poly-methyl-methacrylate denture base materials does not significantly improve transverse bend or impact strength compared to conventional heat-cured acrylic resins. Considering the use of 5 wt%, 15wt% and 20wt% aluminium oxide, copper and silver powder in recent studies this study was performed using 5 wt% nanosilver in-orders to minimize the probable adverse effects on the mechanical and chemical proper-ties of the acrylic base. We found unfavorable brownish discoloration of the dentures to be the most significant problem with this low percentage of nanosilver powder. The other reason for choosing such low percentage of nanosilver was to significantly lower the patient costs for the dentures containing nanosilver particles. Adding 5 wt% of nanosilver powder to poly-methyl-methacrylate improved its thermal conductivity. This improvement was probably due to the uniform distribution of metal particles within the PMMA. To reach this goal, the nanosilver and acrylic powders were poured into an amalgamator mixer to mix the fine particles and produce a uniform mixture. In similar studies adding 5 wt% of silver, copper and aluminium to poly-methyl-methacrylate im-proved its thermal conductivity, although it decreased its tensile strength.

## Limitations of the study:

Incorporation of titanium dioxide by ultrasonic dispersion showed restriction of homogenous dispersion of the nanoparticles. Other methods of incorporation are mortar and pestle, high energy ball milling and silination of which ball milling seems to be the most effective. Future experimentation will add ress the above limitations and will investigate an easier method of incorporation. The lack of homogenous dispersion is also attributed to the morphology of the nanoparticles. Nanoparticles like nanotubes and nanorods show more surface area and hence might result in better bonding to the surface of the Poly methyl methacrylate.

## Conclusion:

Results showed that the injection molded group out-performed the compression molded group in terms of flexural load values with the unmodified control groups exhibiting the highest flexural strength. The injection molded group showed the highest mean flexural strength of 119±8.859. However, a significant decline in flexural strength was observed as titanium dioxide nanoparticle concentration increased. This shows a negative correlation between nanoparticle concentration and flexural strength. This further suggests that the addition of nanoparticles may compromise the material's mechanical properties and their use should be carefully optimized to achieve desired outcomes.

## Figures and Tables

**Table 1:** Flexural load values

**Nc**	**N0.5**	**N1**	**IMC**	**IM0.5**	**IM1**
156	147	150	186	176	137
146	154	127	179	168.3	166
174	136	147	182	175.6	171
168	147	140	194	189.3	135
143	115	137	178	195.2	148
124	126	123	157	176	158
145	137	117	163	147.2	159
154	143	109	167	159.3	164
146	149	119	178	173.4	167
148	157	123	181	167.6	144
153	157	138	198	170.3	140
145	129	137	188	183.5	157
148	147	129	160	147.4	143
154	149	143	178	162.9	139
145	139	119	180	159.3	156
140	137	108	198	175.3	129
138	132	153	190	146.8	170
164	145	128	159	155.8	176
121	147	119	179	167.9	144
124	124	135	201	175.4	133

**Table 2:** Flexural strength values

**S.no**	**Compression molding technique**			**Injection molding technique**		
**Subgroup**	**Nc**	**N0.5**	**N1**	**IMc**	**IM0.5**	**IM1**
1	104.27	98.27	99.67	124	117.3	91.53
2	97	102.4	84.47	119.47	112.2	110.5
3	115.67	90.53	98	121.33	117.1	114.3
4	111.93	97.87	93	129.33	126.2	89.67
5	95.47	76.8	91.07	118.4	130.1	98.6
6	82.53	84	82.07	104.67	117.3	105.6
7	96.4	91.33	78.2	108.93	98.13	106.1
8	102.33	95.53	72.67	111.6	106.2	109.2
9	97.2	99.6	79.53	118.87	115.6	111.6
10	98.6	104.9	82	120.53	111.7	95.8
11	102.07	104.9	91.87	132.2	113.5	93
12	96.93	86.2	91.13	125.6	122.3	104.5
13	98.33	98.27	86	106.53	98.27	95.33
14	102.47	99.33	95.47	118.67	108.6	92.93
15	96.4	92.47	79.33	120.2	106.2	104.3
16	93.07	91.47	71.87	132.27	116.9	86.27
17	91.93	88.07	102.3	126.87	97.87	113.1
18	109.53	96.87	85	106.27	103.9	117.1
19	80.8	97.67	79.33	119.2	111.9	95.73
20	82.53	82.4	90.13	134.27	116.9	88.4

**Table 3:** Analysis of variance test

**Subgroup**	**No of samples**	**Mean±SD**	
Nc	20	97.77±9.128	F-value=8.844; p=0.000P<0.01
N0.5	20	93.94±7.601	
N1	20	86.65±8.685	
IMc	20	119.96±8.859	F-value=21.53; p=0.000p<0.01
IM0.5	20	112.42±8.900	
IM1	20	101.17±9.559	
